# Supplementary material for: The gut microbiota is essential for Trichinella spiralis—evoked suppression of colitis
Source: PLoS Negl Trop Dis. 2024 Nov 4;18(11):e0012645. doi: 10.1371/journal.pntd.0012645 (PMC11563474; doi:10.1371/journal.pntd.0012645)
Supplement: S1 Table — (DOCX) [file pntd.0012645.s001.docx]

**S1 Table Primers used for RT-qPCR analysis**

| Genes | Primer | Sequence（5’-3’） |
| --- | --- | --- |
| IL-10 | Forward | CCCTTTGCTATGGTGTCCTT |
|  | Reverse | TGGTTTCTCTTCCCAAGACC |
| IL-β | Forward | AGCTCTCCACCTCAATGGAC |
|  | Reverse | ATCATTGCGTGGGATCTTGA |
| IL-6 | Forward | TAGTCCTTCCTACCCCAATTTCC |
|  | Reverse | TTGGTCCTTAGCCACTCCTTC |
