# Supplementary material for: The gut microbiota is essential for Trichinella spiralis—evoked suppression of colitis
Source: PLoS Negl Trop Dis. 2024 Nov 4;18(11):e0012645. doi: 10.1371/journal.pntd.0012645 (PMC11563474; doi:10.1371/journal.pntd.0012645)
Supplement: S1 Fig — Stool consistency (A) and occult blood in the stool (B) were detected daily in mice that received ABX treatment but did not receive DSS treatment. Mice present ABX treatment during the induction of colitis exhibits an earlier onset of (C) diarrhea and (D) rectal bleeding compared to mice without ABX treatment. The data shown are means ± SD. Representative results from one out of two independent experiments with n = 5. *P < 0.05 compared to DSS group. ABX: antibiotic treated; Ts, T. spiralis infected; ABXTs: T. spiralis infected and antibiotic treated; DSS: DSS- induced colitis; ABX-DSS: antibiotic- treated and DSS- induced colitis (DOC) [file pntd.0012645.s002.doc]

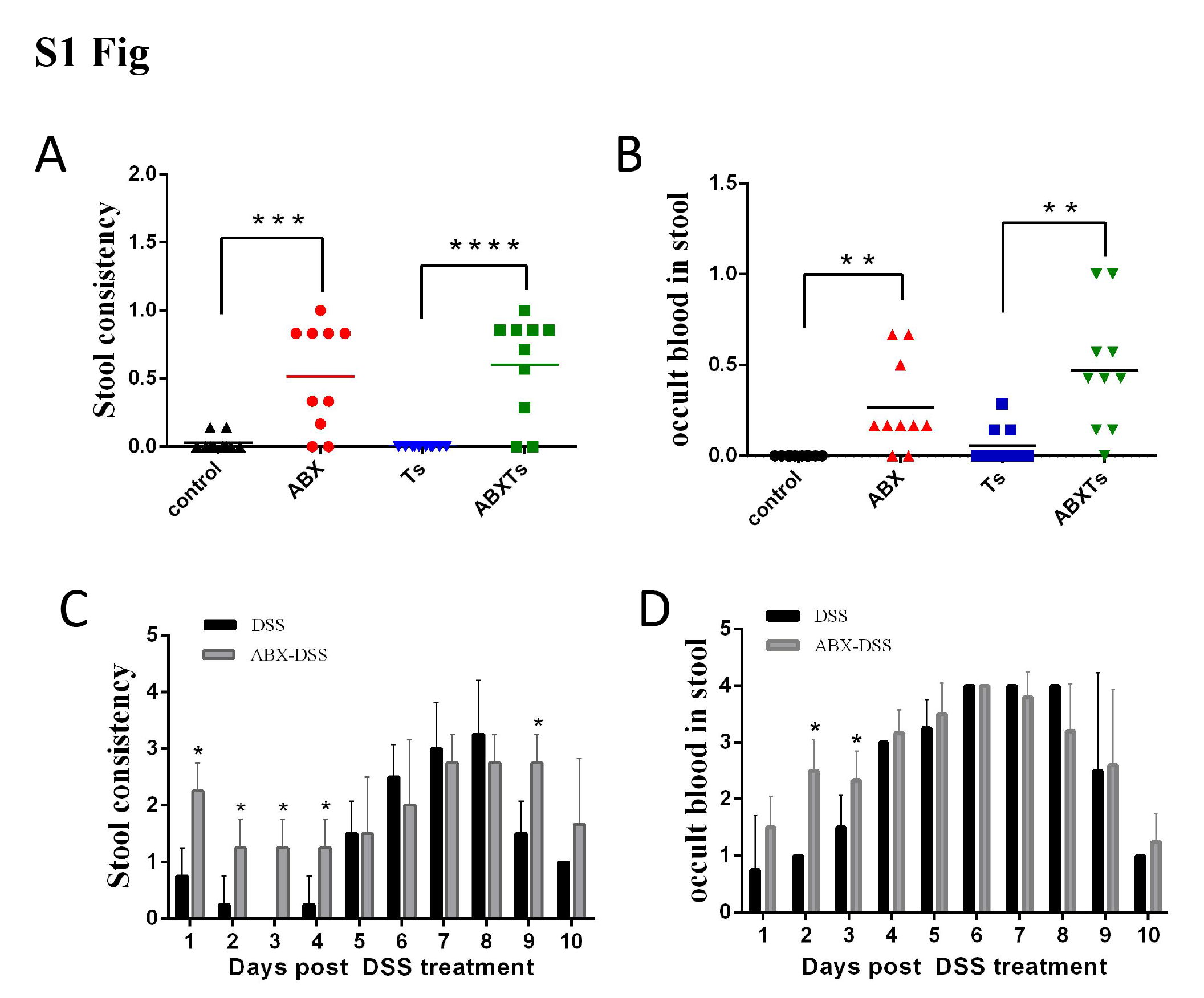
**S1 Fig.** **ABX treatment induces a state of mild intestinal inflammation in mice.** Stool consistency (**A**) and occult blood in the stool (**B**) were detected daily in mice that received ABX treatment but did not receive DSS treatment. Mice present ABX treatment during the induction of colitis exhibits an earlier onset of (**C**) diarrhea and (**D**) rectal bleeding compared to mice without ABX treatment. The data shown are means ± SD. Representative results from one out of two independent experiments with n = 5. **P* < 0.05 compared to DSS group. ABX: antibiotic treated; Ts, *T. spiralis* infected; ABXTs: *T. spiralis* infected and antibiotic treated; DSS: DSS- induced colitis; ABX-DSS: antibiotic- treated and DSS- induced colitis
