# Supplementary material for: The gut microbiota is essential for Trichinella spiralis—evoked suppression of colitis
Source: PLoS Negl Trop Dis. 2024 Nov 4;18(11):e0012645. doi: 10.1371/journal.pntd.0012645 (PMC11563474; doi:10.1371/journal.pntd.0012645)
Supplement: S2 Fig — (A) Weight change in percent. (B) The changes in DAI, scored from diarrhea, bleeding and body weight loss. (C) The histopathological changes were examined by H&E staining, the black bar indicates 200 μm. (D) Histopathological scores. The data shown are means ± SD. Representative results from one out of two independent experiments with n = 4. Ts, T. spiralis- infected; coTs: T. spiralis- infected mice cohoused with normal mice; Ts-DSS: T. spiralis- infected and DSS- induced colitis; coTs-DSS: T. spiralis- infected, cohoused with normal mice and DSS- induced colitis (DOC) [file pntd.0012645.s003.doc]

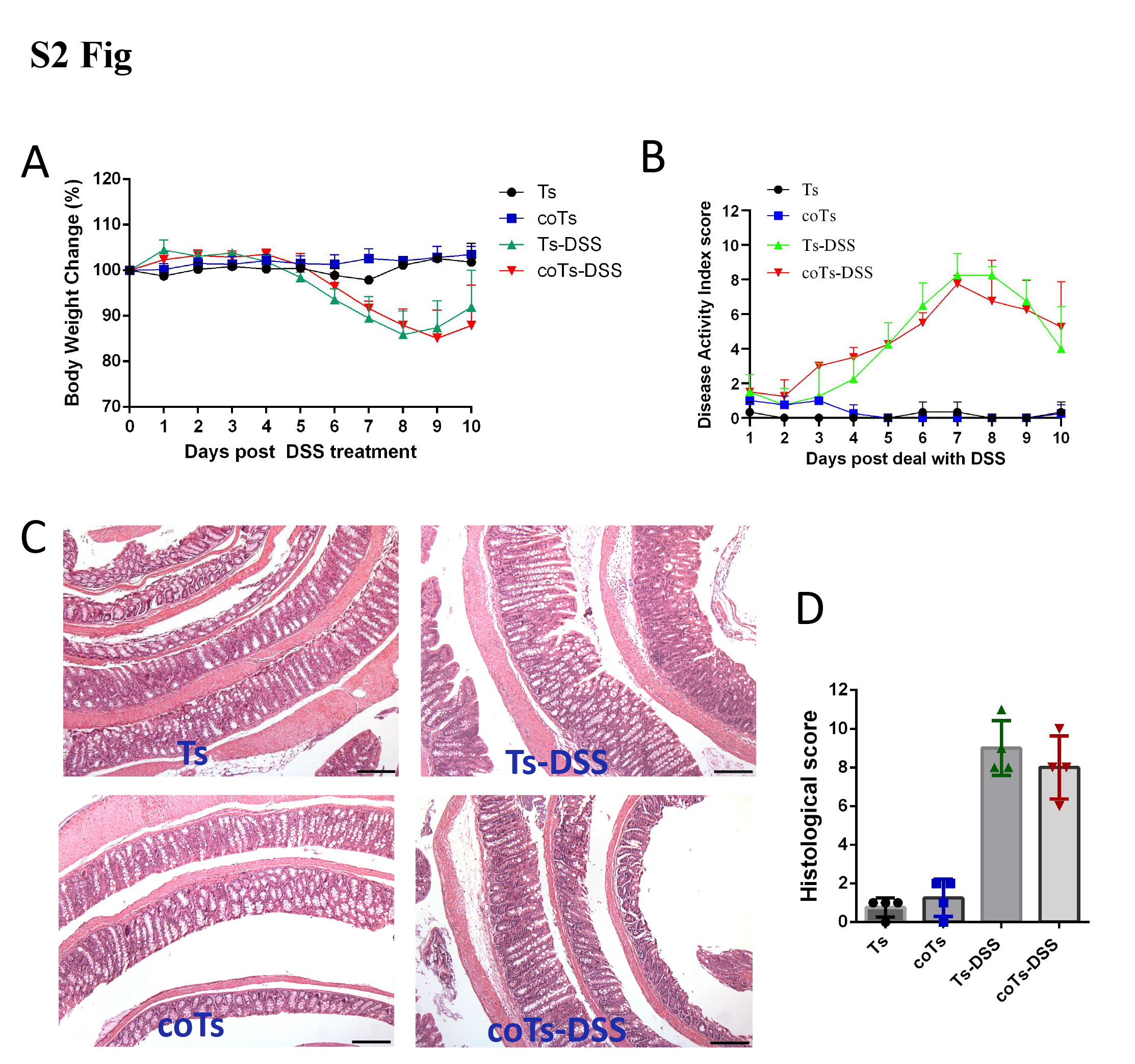
**S2 Fig. Cohousing has no impact on the DSS-induced colitis in mice present *T. spiralis* infection.** (**A**) Weight change in percent. (**B**) The changes in DAI, scored from diarrhea, bleeding and body weight loss. (**C**) The histopathological changes were examined by H&E staining, the black bar indicates 200 μm. (**D**) Histopathological scores. The data shown are means ± SD. Representative results from one out of two independent experiments with n = 4. Ts, *T. spiralis-* infected; coTs: *T. spiralis-* infected mice cohoused with normal mice; Ts-DSS: *T. spiralis-* infected and DSS- induced colitis; coTs-DSS: *T. spiralis-* infected, cohoused with normal mice and DSS- induced colitis
