# Supplementary material for: The gut microbiota is essential for Trichinella spiralis—evoked suppression of colitis
Source: PLoS Negl Trop Dis. 2024 Nov 4;18(11):e0012645. doi: 10.1371/journal.pntd.0012645 (PMC11563474; doi:10.1371/journal.pntd.0012645)
Supplement: S3 Fig — (A) Alpha diversity analysis (Chao 1 and ACE index), data are shown as median, maximum, minimum, upper quartile and lower quartile. (B) Beta diversity analysis (PCoA based on Binary-Jaccard). The relative abundance of the top 10 phyla (C) and the top 20 genus (D) in cohousing control mice and cohousing T. spiralis- infected mice. (E) Cladogram was obtained from the LEfSe analysis when the effect size threshold of LDA was set to 3.5. cocontrol: normal mice cohoused with T. spiralis -infected mice; coTs: T. spiralis -infected mice cohoused with normal mice (DOC) [file pntd.0012645.s004.doc]

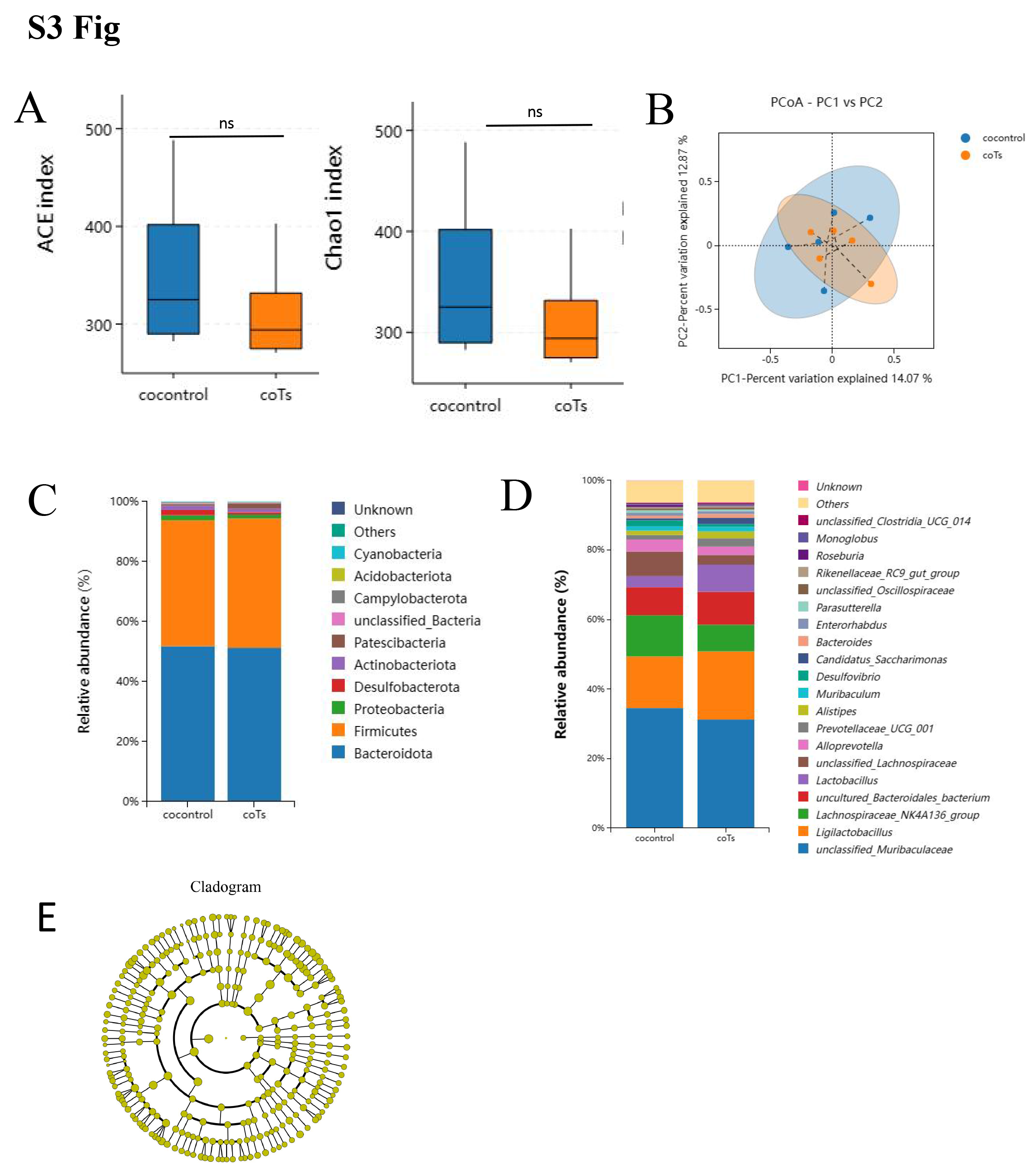
**S3 Fig. The gut microbiota composition in mice becomes consistent when cohousing of control and *T. spiralis*- infected mice.** (**A**) Alpha diversity analysis (Chao 1 and ACE index), data are shown as median, maximum, minimum, upper quartile and lower quartile. (**B**) Beta diversity analysis (PCoA based on Binary-Jaccard). The relative abundance of the top 10 phyla (**C**) and the top 20 genus (**D**) in cohousing control mice and cohousing *T. spiralis*- infected mice. (**E**) Cladogram was obtained from the LEfSe analysis when the effect size threshold of LDA was set to 3.5. cocontrol: normal mice cohoused with *T. spiralis* -infected mice; coTs: *T. spiralis* -infected mice cohoused with normal mice
